# Influence of the Size of the Fiber Filler of Corn Stalks in the Polylactide Matrix Composite on the Mechanical and Thermomechanical Properties

**DOI:** 10.3390/ma14237281

**Published:** 2021-11-28

**Authors:** Daniel Łączny, Marek Macko, Krzysztof Moraczewski, Zbigniew Szczepański, Andrzej Trafarski

**Affiliations:** 1 Faculty of Mechatronics, Kazimierz Wielki University, Chodkiewicza 30, 85-064 Bydgoszcz, Poland; mackomar@ukw.edu.pl (M.M.); zszczep@ukw.edu.pl (Z.S.); 2 Institute of Material Engineering, Kazimierz Wielki University, Chodkiewicza 30, 85-064 Bydgoszcz, Poland; kmm@ukw.edu.pl (K.M.); trafarski@ukw.edu.pl (A.T.)

**Keywords:** polylactide, green composites, maize stalk, mechanical properties

## Abstract

This paper presents results of a study on the effect of filler size in the form of 15 wt% corn stalk (CS) fibers on the mechanical and thermomechanical properties of polylactide (PLA) matrix composites. In the test, polylactidic acid (PLA) is filled with four types of length of corn stalk fibers with a diameter of 1 mm, 1.6 mm, 2 mm and 4 mm. The composites were composed by single screw extrusion and then samples were prepared by injection molding. The mechanical properties of the composites were determined by static tensile test, static bending test and Charpy impact test while the thermo-mechanical properties were determined by dynamic mechanical thermal analysis (DMTA). The composite structures were also observed using X-ray microcomputed tomography and scanning electron microscopy. In the PLA/CS composites, as the filler fiber diameter increased, the degradation of mechanical properties relative to the matrix was observed including tensile strength (decrease 22.9–51.1%), bending strength (decrease 18.9–36.6%) and impact energy absorption (decrease 58.8–69.8%). On the basis of 3D images of the composite structures for the filler particles larger than 2 mm a weak dispersion with the filler was observed, which is reflected in a significant deterioration of the mechanical and thermomechanical properties of the composite. The best mechanical and thermomechanical properties were found in the composite with filler fiber of 1 mm diameter. Processing resulted in a more than 6-fold decrease in filler fiber length from 719 ± 190 µm, 893 ± 291 µm, 1073 ± 219 µm, and 1698 ± 636 µm for CS1, CS1.6, CS2, and CS4 fractions, respectively, to 104 ± 43 µm, 123 ± 60 µm, 173 ± 60 µm, and 227 ± 89 µm. The fabricated green composites with 1 to 2 mm corn stalk fiber filler are an alternative to traditional plastic based materials in some applications.

## 1. Introduction

The properties of polymer materials such as low density, high chemical resistance, barrier properties, high mechanical strength, and ease of processing have led to their use in many everyday products [1,2,3]. In 2019, 367 million tons of polymer plastics were produced globally of which more than 90% are obtained from fossil resources [4]. Problems regarding environmental pollution with their waste and the declining stocks of fossil fuels from which most of them are produced resulted in many studies on total or partial replacement of synthetic polymeric materials with natural and renewable ones [5,6,7,8]. In addition, modern society and international legislation promotes the production of sustainable materials and the circular model, in which green composites fit well [9,10].

Green composites usually consist of biodegradable and/or bio-based polymer matrix and natural organic fibers/particles as reinforcement or filler [11,12]. They are characterized by lower production cost, biodegradability, aesthetic and more “natural” appearance, as well as often inferior mechanical properties compared to synthetic composites [13]. Over the past years, studies have been conducted on a wide range of biodegradable polymer matrices obtained from both natural and petrochemical sources [13,14]. Among the polymer matrices used in green composites, polylactide (PLA) is very popular. It belongs to the group of bioplastics, i.e., polymers produced both from natural resources and biodegradable to water, carbon dioxide, and biomass within a few to several weeks [15]. The PLA is a thermoplastic aliphatic polyester produced from the polymerization of lactic acid (LA), which is derived from the fermentation of renewable plant-based raw materials such as corn, potato, rice, and soy [16]. It is classified as a sustainable material because it is possible to compost it and further use its decomposition products as fertilizer in agricultural production [17]. The PLA is a polymer with a high tensile strength of 70 MPa and an elastic modulus of 3 GPa. This polymer can be recycled 7 to even 10 times [18]. The disadvantages of PLA include high brittleness, high flammability, poor thermal stability, and poor barrier properties [19]. Therefore, materials are often added to the PLA matrix in order to improve the polymer properties [20], including various lignocellulosic fibers such as bamboo [21], *Opuntia ficus-indica* and *Posidonia oceanica* [12,22], wood [23] and hemp [24].

The fibers/particles that provide reinforcement or filler roles in the green composite can be of plant or animal origin. Natural fibers, compared to mineral fibers, have lower density, biodegradability, reduced health risks, lower purchase cost, and lower abrasiveness to prevent equipment damage. The main disadvantages of natural fibers are poor filler compatibility, hygroscopicity, inconsistency of fibers, poor thermal stability, low durability and variability of properties depending on processing methods, fiber type, environmental conditions and modification [25,26]. The mechanical properties of composites reinforced with fibrous fillers are affected by the distribution of fiber in the composite structure, their size including diameter and length, thermal and chemical stability of the matrix, fiber strength and elasticity, and the nature of the fiber–matrix interface [27]. Fiber modification is often conducted to improve the bonding in the composite between the hydrophilic filler and the hydrophobic polymer matrix resulting in, among others, an increase in the mechanical properties of the composite [28,29,30]. Fillers from agricultural, forestry, or food industry by-products have a smaller carbon footprint, [31] while not increasing competition for food [32].

Corn stalks are one of the wastes generated from the cultivation of corn grains, which plays a significant role in the agri-food industry and is a fundamental food ingredient in many diets around the world [33]. In 2019, 1116 million tons of corn grain was obtained and production is expected to increase by 20% in 20 years [34]. Each 1 kg of dry corn grain yielded produces about half a kilogram of corn stalks and half a kilogram of other types of crop residues including leaves, cobs and husks, more than 90% of which are left in the field or are burned [35,36]. The corn stalk is a non-homogeneous material consisting of skin and flesh. The skin is characterized by high hardness and tensile strength, while the flesh has low density and good thermal insulation properties [37,38]. Corn stalks can be used to produce biogas, biocarbon, and biomethane [39,40,41]. Chipboard [42], insulation boards, and polymer composites [43,44] can be produced from them. Corn stalk fibers, both chemically modified [45] and unmodified [46,47], are used in polymer composites. Despite their high availability, low purchase price and good mechanical properties, corn stalks are rarely used as a filler for biodegradable polymers. The target fiber size of corn stalks used in polymer composites is usually obtained on shredders and knife mills [45,46,48]. The shape and size of the fiber used in the composite affect its final properties [12,22,49]. There are no studies analyzing the effect of corn stalk fiber size in polylactide matrix composites before and after processing on mechanical and thermomechanical properties.

The purpose of this paper was to investigate the effect of corn stalk fiber size on the mechanical (tensile strength, bending strength, impact strength) and thermomechanical properties of corn stalk/polylactide composites prepared by single screw extrusion and injection molding. In the present study, corn stalks collected from the surrounding areas were only subjected to drying, shredded on a single disc shredding system, separated into fractions and then blended with polylactide. Fiber size analysis of corn stalks before processing by optical microscopy and after processing by scanning electron microscopy was performed. In addition, the fiber distribution in the composite was analyzed using computer microtomography. An innovation of presented research was the possibility of testing biodegradable polylactide composites containing natural fillers with the best possible mechanical properties only by choosing an appropriate filler size and not by chemical modification of the fibers as it has been used so far. This will allow to obtain good biodegradable composites without degrading their biodegradation properties, which could be reduced by adding synthetic chemicals on the modified fibers.

## 2. Materials and Methods

### 2.1. Materials

The PLA polylactide Inego 3251D from (NatureWorks, Minnetonka, MN, USA) with a density of 1.24 g/cm^3^, molecular weight of 55.4 g/mol, and MFI of 35 g/10 min (190 °C/2.16 kg), which is dedicated to injection molding applications, was used for this study. From whole corn plants 31.255 (Limagrain, Mogilno, Poland) harvested manually by cutting 10 cm above the ground, corn stalks were separated and then dried at 18 °C for 1450 h.

### 2.2. Shredding and Screening of the Corn Stalk

**Figure 1:**
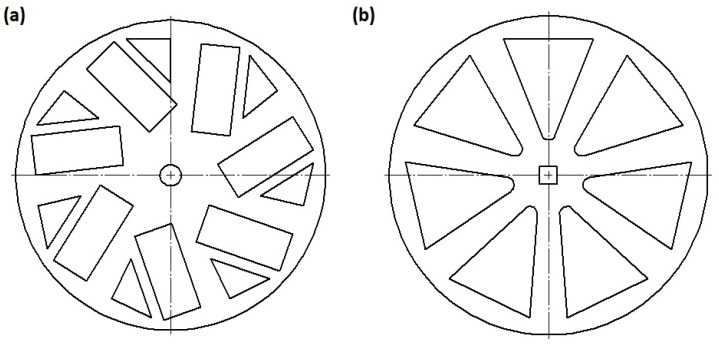
View of the disks of shredding system: (**a**) stationary disk, (**b**) moving disc.

Shredding of corn stalks was carried out on a ULR-2.0/2004 universal laboratory shredder (Bydgoszcz, Poland) equipped with a disc-shredding system operating at a peripheral speed of the moving disc of 16.5 m/s. The moving disc had triangle-shaped holes and the stationary disc had rectangular and triangle-shaped holes, as shown in Figure 1.

The shredded material was screened using an Anlysette 3 Pro sieve analyzer (Fritsch, Idar-Oberstein, Germany). Five DIN-ISO 3310/1 measuring sieves (Retsch, Eragny, France) with mesh sizes of 4 mm, 2 mm, 1.6 mm, 1 mm, and 0.8 mm were used for screening. The material was sieved in batches of 50 g for a period of 10 min at an amplitude of 2.5 Hz. A 120 g fraction obtained on each of the 2 mm, 1.6 mm, 1 mm, and 0.8 mm mesh sieves was used for further testing due to the higher mass proportion of corn stalk skin in these fractions [43].

### 2.3. Preparation of Samples

Based on previous studies on the effect of filler amount of shredded corn stalks in PLA matrix composites on mechanical properties, it was determined that the composite samples in this study would contain 15 wt% filler amount [46,50]. The PLA granules and fractions of shredded corn stalks were dried in a SUP-100 laboratory dryer (Wamed, Warsaw, Poland) at 40 °C for 48 h to remove moisture. The materials were then weighed using an analytical balance WAS 160/X (Radwag, Radom, Poland) and composite formulations were prepared containing 15 wt% filler from each fraction of shredded corn stalks. The composite formulations were designated as PLA/CS4, PLA/CS2, PLA/CS1.6, and PLA/CS1, respectively. The manually mixed composite formulations were poured into the hopper of a W25-30D single-screw extruder (Metalchem, Gliwice, Poland). The extrusion temperature profile was identical for all compositions (1st section, 150 °C; 2nd section, 155 °C; 3rd section, 155 °C; head, 155 °C). The screw speed was set at 30 rpm. The sample containing pure polylactide was extruded under identical conditions as the composite formulations. The resulting extrudates were cooled with compressed air and granulated using a knife granulator. The obtained granules from each composition were dried in a SUP-100 laboratory dryer (Wamed, Warsaw, Poland) at 40 °C for 48 h and then dumbbell-shaped samples were produced by injection molding on a TRX 80 Eco injection molding machine (Tederic, Shanqhai, China). The injection molding process for all compositions was conducted under the following conditions: 1st screw section temperature—170 °C, 2nd screw section temperature—170 °C, 3rd screw section temperature—175 °C, injection temperature—180 °C, mold temperature—35 °C, injection pressure—248 bar, cooling time—60 s.

### 2.4. Characteristics of PLA and Composites

The density was determined using MVP-D160E pycnometer (Quantachrome Instruments, Ashland, VA, USA) according to the PN-EN ISO 1183 standard [51]. Helium was used in the study. The density value was calculated as the arithmetic mean of 2 measurements.

For mechanical tensile, bending, and impact tests, ten samples from each composition were used per test. The bending and tensile tests of samples were carried out on an Instron 3367 testing machine (Instron, Norwood, MA, USA) at room temperature of 20 ± 2 °C and Charpy impact tests were carried out on unnotched samples using an XJ 5Z impact hammer (Liangong, Shandong, China) at the same temperature. Static tensile test was carried out according to EN ISO 527 [52] with a tensile speed of 50 mm/min. Static bending test was carried out according to EN ISO 14125 [53], where the support spacing was 64 mm and the bending speed was 20 mm/min. Impact tests were carried out according to PN-EN ISO 179-2 [54] using a hammer of 2 J. The results are the arithmetic mean of each test.

The effect of temperature on the mechanical properties of PLA and PLA/CS samples was investigated by dynamic mechanical analyzer type Q800 (TA Instruments, New Castle, DE, USA) according to PN-EN ISO 6721 [55]. Tests were carried out in the temperature range from 25 to 150 °C with a heating rate of 3 °C/min. Samples were cuboid in shape with dimensions of 80 mm × 10 mm × 4 mm. The deformation strain was 0.01% and the frequency was 1 Hz.

Observations of the corn stalk fiber surface of each fraction obtained from sieve analysis were made using a DMS-350 digital microscope (Leica Microsystems, Heerbrugg, Switzerland), and the resulting microphotographs were analyzed in Leica Application Suite LAS 4.12 software.

Observations of the composite structure were conducted using Skyscan 1273 X-ray microtomography (Bruker, Karlsruhe, Germany) and scanning electron microscopy (SEM) with a Phenom XL microscope (Thermo Fisher Scientific, Waltham, MA, USA). The microtomography study was conducted with the following parameters: scaled image pixel size, 4.9 µm; exposure time, 570 ms; voltage, 50 kV; and current, 50 µA. The samples for microtomography were cut from the center of standard paddles and had dimensions of 10 mm × 4 mm × 30 mm. The SEM test was carried out at an accelerating voltage of 5 kV in a high vacuum of 0.1 Pa. The surface of the test samples for SEM was coated with a layer of gold in an MCM-100P low vacuum sputtering machine (Sec, Gyeonggi-do, Korea) for 30 s to avoid electrostatic discharge during the test. The surface observations of samples were conducted in mapping mode, with a BSD Full (Back Scatter Detector).

## 3. Results

### 3.1. Density

The measured density of pure PLA granules was 1.2562 ± 0.0013 g/cm^3^. The obtained value differed slightly from the producer’s reported density [56]. The composites produced had a lower density than pure PLA (Table 1) due to the lower density of the CS filler relative to the matrix. Ma et al. observed that the CS fiber content in the phenolic resin matrix polymer composite also resulted in a decrease in the density of the composite produced [44]. The density differences between the composites studied may have been influenced by the content of various components of the corn stalk including the skin and flesh. The skin has a higher density than the flesh [43].

### 3.2. Characteristics of Mechanical Properties

**Figure 2:**
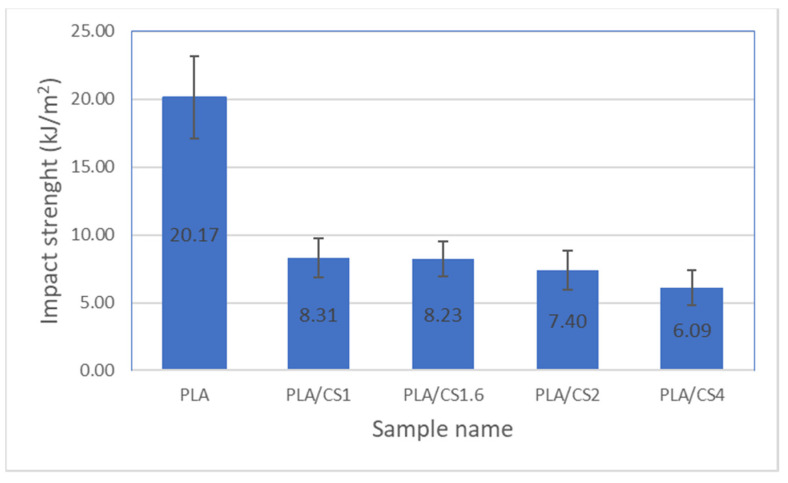
Impact strength of PLA and its composites.

Figure 2 shows the effect of corn stalk fiber size on the impact energy of produced samples. The samples containing pure PLA absorbed the impact energy best. As the filler fiber size increases, the impact energy decreases from 8.31 ± 1.64 kJ/m^2^ for PLA/CS1 samples up to 6.09 ± 1.08 kJ/m^2^ for PLA/CS4 samples which may be related to the deterioration of fiber dispersion with fiber length. A similar correlation was observed by Golmakani et al. where the impact energy decreased with increasing wood flour size in the polyethylene matrix composite [57].

The tensile and bending properties of injection molded samples of pure PLA and PLA/CS composites are shown in Table 2 and Table 3, respectively.

**Figure 3:**
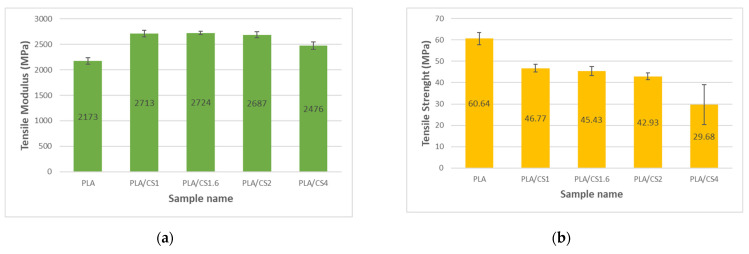
Impact of corn stalk fiber size on tensile (**a**) modulus; (**b**) strength.

**Figure 4:**
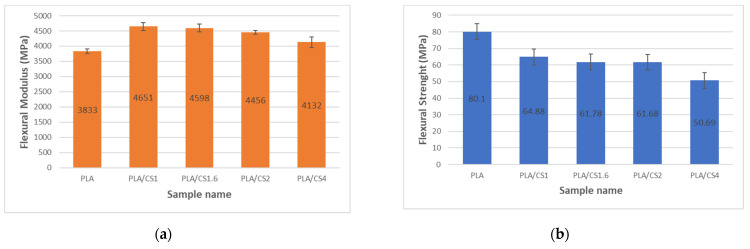
Impact of corn stalk fiber size on flexural (**a**) modulus; (**b**) strength.

The PLA samples showed the lowest elastic modulus in both tension (Figure 3a) and bending (Figure 4a) of 2173 ± 65 MPa for tension and 3833 ± 71 MPa for bending, respectively. The use of CS fiber resulted in an increase in tensile and bending modulus in the composites relative to the matrix which is also observed with other types of natural fibers [58]. The highest tensile modulus of elasticity amounting to 2724 ± 34 MPa was for the PLA/CS1.6 sample, while the highest bending modulus of elasticity amounting to 4651 ± 129 MPa was for the PLA/CS1 sample. For the PLA/CS4 samples, in which the filler particles had a diameter of 4 mm, a significant decrease in modulus of elasticity both in tension and bending can be observed in relation to the other composites with CS filler. Similar decrease in tensile and bending modulus values in polymer composites with fibrous fillers for particles with diameter over 2 mm was observed by Stanciu et al. [59].

**Figure 5:**
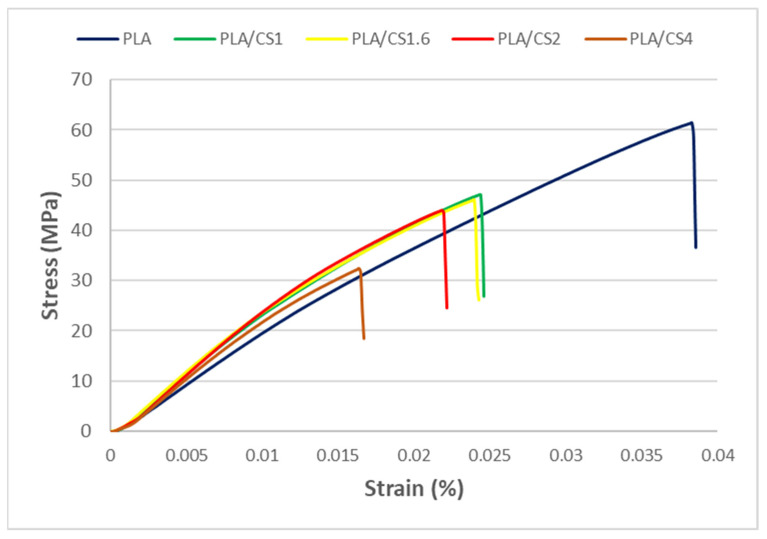
Stress–strain curve in tensile tests of pure PLA and PLA/CS composite samples.

The highest tensile strength (Figure 3b) as well as bending strength (Figure 4b) of pure PLA samples were 60.64 ± 2.82 MPa for tension and 80.01 ± 2.3 MPa for bending, respectively. The value of tested PLA tensile strength was slightly different from that reported by the producer [56]. The tensile and bending strengths of the CS composites decreased with increasing fiber diameter from 46.77 ± 1.73 MPa for PLA/CS1 to 29.68 ± 9.33 MPa for PLA/CS4 in tension and from 64.88 ± 2.5 MPa for PLA/CS1 to 50.69 ± 5.3 MPa for PLA/CS4. However, there is a noticeable slight difference between the obtained tensile and bending strength values for filler particles in the range of 1 to 2 mm and a large decrease in strength for particles above 2 mm. Figure 5 shows the stress–strain curves for tensile tests of pure PLA and PLA/CS composite samples.

Similar correlation as for tensile and bending modulus and strength is also observed for tensile and bending strain. The pure PLA samples have the highest tensile strain and bending strain values of 4 ± 0.7% for tension and 2.24 ± 0.42% for bending, respectively. The use of CS fiber reduced the strain to about 1.53–2.34% for tensile and 1.6–1.79% for bending, which is a frequently observed effect of lignocellulosic particle filler [60].

Deterioration of the mechanical properties of PLA/CS composites relative to pure PLA is related to the poor interfacial adhesion occurring between the hydrophilic lignocellulosic CS fiber and the hydrophobic polymer matrix. The weak interfacial adhesion results in poor stress transfer between the composite components [60,61]. As the fiber diameter increases, the contact area with the matrix increases which causes an increase in stress intensification points and defects leading to the formation of sites for crack initiation causing sample failure even at lower loading values. Nourbachsh et al. indicated that a greater number of filler fiber–matrix contact areas in smaller particles results in improved tensile strength [62]. It is also possible that as the lignocellulosic material particles decrease, there is better and more uniform mixing with the polymer matrix as well as better flow through the injection hole resulting in better mechanical properties [63,64].

Improvement of interfacial adhesion in lignocellulosic filler composite is possible by using pretreatment of filler fibers including corn stalks. Luo et al. showed that pretreatment with epoxy emulsion modified with isocyanates of corn fibers used as a filler in PLA matrix composite allows to increase the interfacial adhesion between composite components, which results, among others, in improvement of mechanical properties [45].

### 3.3. Characteristics of Thermomechanical Properties

**Figure 6:**
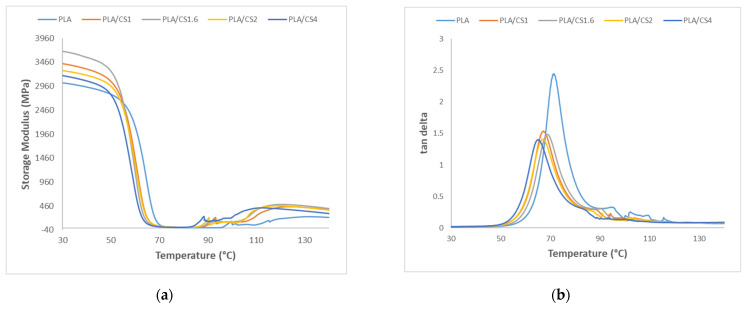
Change of (**a**) storage modulus and (**b**) dynamic damping factor (tan δ) as a function of the temperature of pure PLA and PLA/CS composite samples.

The viscoelastic properties of pure PLA and PLA/CS composite samples have been investigated by dynamic mechanical analysis (DMA) method with measurement of the change in the conservation modulus (G′) and damping ratio (tan δ) as a function of temperature, as shown as curves in Figure 6. The variation of conservative modulus E′ with increasing temperature, shown in Figure 6a, indicates that at low temperatures below 50 °C, a relatively high stiffness is observed for all tested samples, which decreases quickly at temperatures between 50 °C and 80 °C due to reaching the alpha (α)-relaxation temperature of the polymer. On the other hand, in the temperature range between 100 and 140 °C, the phenomenon of cold crystallization is observed.

The maximum value of tan δ and the temperature at which this value is obtained as well as the values of E′ measured at 30 °C, 50 °C, 75 °C, and 120 °C for the samples of pure PLA and PLA/CS composites are presented in Table 4. All PLA/CS composites showed higher E′ values than pure PLA. The largest difference is observed after the glass transition area at 75 °C where the E′ value for the PLA/CS2 sample relative to unfilled PLA is 300% higher, indicating a large effect on the E′ value of the hard fillers in the thermoplastic matrix. A similar effect in changing the E′ value of PLA reinforcement with lignocellulosic fibers has been described previously [65,66]. A more than twofold increase in E′ values of 227%, 250%, 240%, and 208% is also observed in PLA/CS composites relative to pure PLA at 120 °C for PLA/CS1, PLA/CS1.6, PLA/CS2, and PLA/CS4, respectively. Additionally, the presence of fibers causes the cold crystallization phenomenon to occur at lower temperatures. The greatest increase in E′ relative to the PLA matrix at 30 °C, 50 °C, 75 °C, and 120 °C occurs in the PLA/CS1 composite.

Figure 6b shows the change in damping ratio (tan δ) as a function of temperature. The peak for the pure PLA sample was at 71.65 °C, while peaks were obtained at slightly lower temperatures for the PLA/CS composite samples. The highest tan δ value of 2.419 was obtained for pure PLA samples while PLA/CS samples had a lower value, which decreased along with increasing fiber length from 1.536 for PLA/CS1 to 1.398 for PLA/CS4. The decrease in tan δ value caused by partial suppression of PLA chain relaxation by CS fibers which consequently results in fewer PLA chains undergoing glass transition [67].

### 3.4. Characteristics of Corn Stalk Fiber Length before Processings

**Figure 7:**
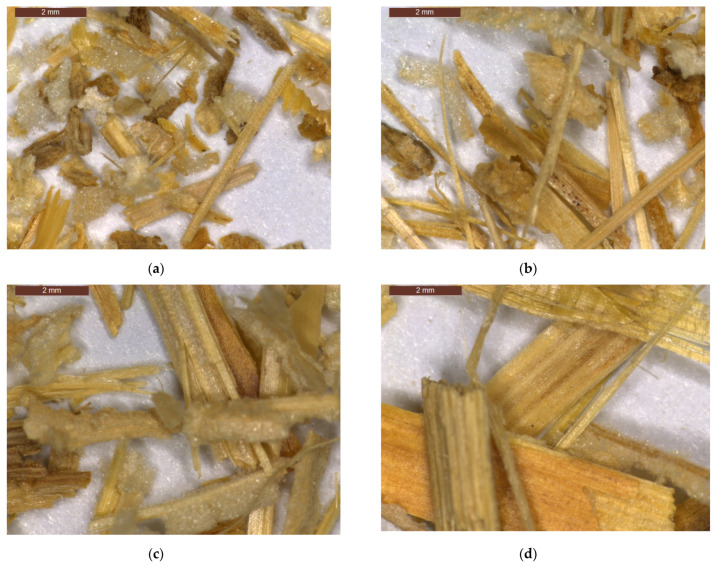
Optical micrographs of corn stalk fiber fractions (**a**) CS1; (**b**) CS1.6; (**c**) CS2; (**d**) CS4.

Figure 7 shows the microphotographs from the optical microscope analysis for each corn stalk fraction. The average corn stalk fiber diameter was 719 ± 190 µm, 893 ± 291 µm, 1073 ± 219 µm, and 1698 ± 636 µm for the CS1, CS1.6, CS2, and CS4 fractions, respectively. The CS1 fraction has the least elongated particles which is reflected in the highest diameter to length ratio value of 0.59 while for the other fractions it was 0.41, 0.44, and 0.36 for CS1.6, CS2, and CS4, respectively. Both components of corn stalks are visible in all fractions, where the intensity of flesh decreases as the size of fraction increases.

### 3.5. Analysis of Fiber Orientation and Size in the Composite

**Figure 8:**
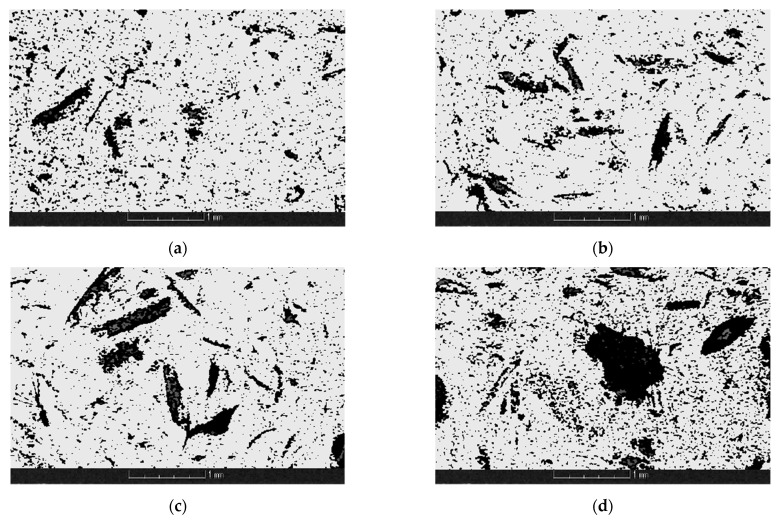
Cross-sections of composites CS: (**a**) PLA/CS1; (**b**) PLA/CS1.6; (**c**) PLA/CS2; (**d**) PLA/CS4.

**Figure 9:**
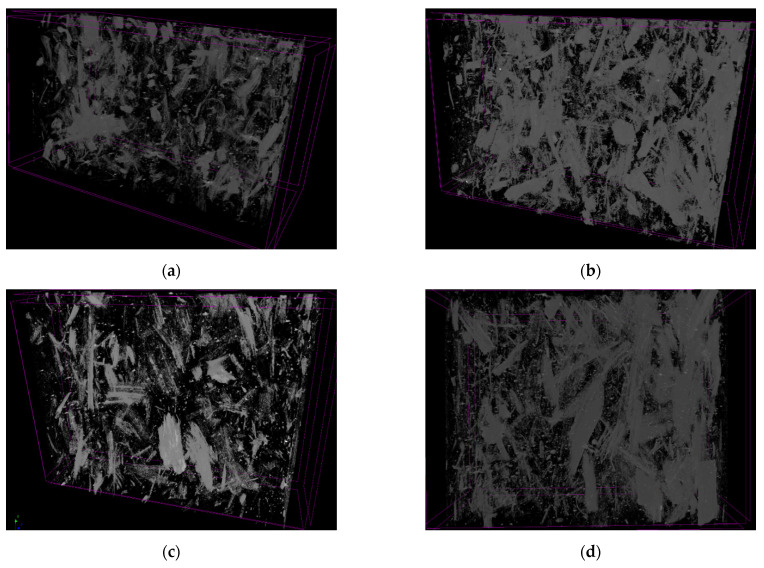
3D images of composites CS: (**a**) PLA/CS1; (**b**) PLA/CS1.6; (**c**) PLA/CS2; (**d**) PLA/CS4.

Images of cross sections of CS composites in a plane parallel to the main direction of material flow in the mold were taken using microtomography. The composite cross sections from the middle section are shown in Figure 8a–d for PLA/CS1, PLA/CS1.6, PLA/CS2, and PLA/CS4 samples, respectively. Based on the obtained composite cross sections, 3D image reconstructions were carried out, which are shown in Figure 9a–d for PLA/CS1, PLA/CS1.6, PLA/CS2, and PLA/CS4 samples, respectively. In all the samples, the CS fibers have a chaotic distribution. Figure 9 shows that in the PLA/CS1, PLA/CS1.6, and PLA/CS2 composites, the CS fibers were uniformly mixed with the binder, while the PLA/CS4 composite shows clusters of CS fibers, which may be responsible for the significant deterioration of mechanical properties of the green composite. In all composites small metal precipitates (≈1 wt%) are observed, most probably due to the disc wear of the grinding system.

**Figure 10:**
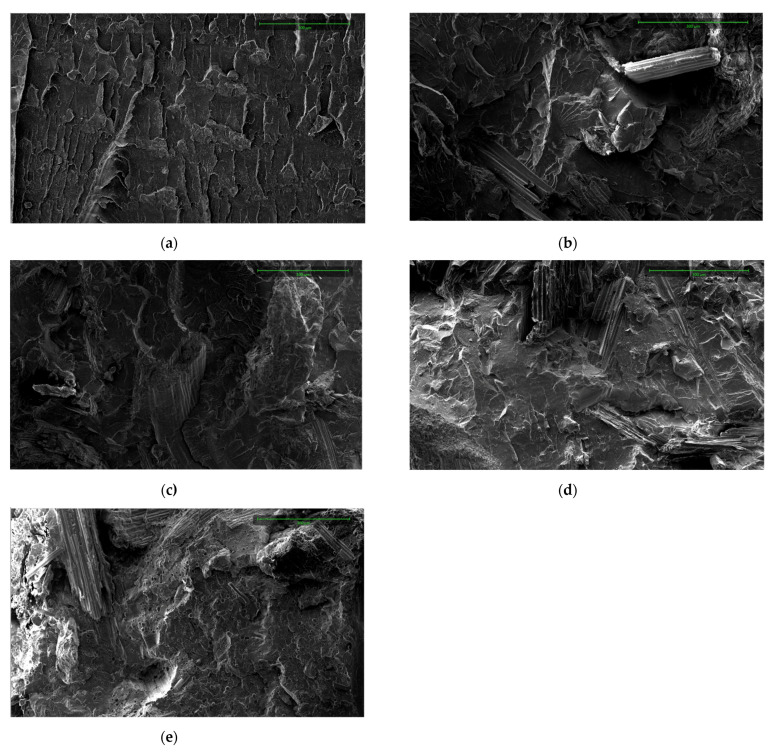
SEM micrographs of composite samples: (**a**) PLA; (**b**) PLA/CS1; (**c**) PLA/CS1.6; (**d**) PLA/CS2; (**e**) PLA/CS4.

Image 10 shows SEM images with 500x magnification of the fracture surface after impact test of pure PLA and PLA/CS composite samples. The observed homogeneous and smooth surface in Figure 3a corresponds to the PLA sample. The gaps between the corn stalk fibers and the polylactide matrix visible in Figure 10b–e indicate poor interfacial adhesion between the fiber and matrix which results in low stress transfer from matrix to filler [68,69] and decrease in mechanical properties. The average fiber diameter observed in SEM is 104 ± 43 µm, 123 ± 60 µm, 173 ± 60 µm, and 227 ± 89 µm for PLA/CS1, PLA/CS1.6, PLA/CS2, and PLA/CS4 samples, respectively. Composite processing resulted in a 693%, 726%, 622%, and 748% decrease in average filler fiber diameter for the sample.

## 4. Conclusions

The mechanical and thermomechanical properties of polymer composites with lignocellulosic fillers are significantly affected by the properties of their components, the quality of bonding between the polymer matrix and the lignocellulosic material, and the homogeneous and uniform distribution of the filler in a composite.

The effect of corn stalk particle size on the mechanical and thermomechanical properties of a polylactide matrix polymer composite was investigated. Corn stalk fiber was used in four sizes—1 mm, 1.6 mm, 2 mm, and 4 mm. It was observed that the mechanical and thermomechanical properties deteriorated with increasing filler size due to better transfer of finer fiber particles of mechanical stresses in the composite. The use of fiber particles larger than 2 mm causes poor dispersion with the polymer matrix, which significantly affects the deterioration of mechanical and thermomechanical properties of the composite. The composite with filler fiber of 1 mm diameter had the best mechanical and thermomechanical properties.

The SEM images indicated that the corn stalk fibers had low interfacial adhesion with the polylactide matrix. Processing resulted in a more than 6-fold decrease in filler fiber length from 719 ± 190 µm, 893 ± 291 µm, 1073 ± 219 µm, and 1698 ± 636 µm for CS1, CS1.6, CS2, and CS4 fractions, respectively, to 104 ± 43 µm, 123 ± 60 µm, 173 ± 60 µm, and 227 ± 89 µm.

In conclusion, corn stalk fiber with a size between 1 and 2 mm has a good potential for their use in the production of green polymer composites compatible with sustainable natural resource management and constitutes an alternative to traditional filler materials.

Further studies will focus on the rheological and structural analysis of the investigated composites.

## Figures and Tables

**Table 1:** Density of tested samples.

Sample Name	Density (g/cm^3^)
PLA	1.2562 ± 0.0013
PLA/CS1	1.2318 ± 0.0025
PLA/CS1.6	1.2134 ± 0.0021
PLA/CS2	1.2489 ± 0.0011
PLA/CS4	1.1995 ± 0.0026

**Table 2:** Tensile properties of tested samples.

Sample Name	Modulus of Elasticity (MPa)	Tensile Strength (MPa)	Strain at Break (%)
PLA	2173 ± 65	60.64 ± 2.82	4 ± 0.7
PLA/CS1	2713 ± 66	46.77 ± 1.73	2.34 ± 0.13
PLA/CS1.6	2724 ± 34	45.43 ± 2.19	2.30 ± 0.17
PLA/CS2	2687 ± 60	42.93 ± 1.66	2.17 ± 0.15
PLA/CS4	2476 ± 74	29.68 ± 9.33	1.53 ± 0.55

**Table 3:** Flexural properties of tested samples.

Sample Name	Modulus of Elasticity (MPa)	Flexural Strength (MPa)	Flexural Break (%)
PLA	3833 ± 71	80.01 ± 10.3	2.24 ± 0.42
PLA/CS1	4651 ± 129	64.88 ± 2.5	1.79 ± 0.19
PLA/CS1.6	4598 ± 136	61.78 ± 3.3	1.76 ± 0.25
PLA/CS2	4456 ± 56	61.68 ± 2.9	1.71 ± 0.15
PLA/CS4	4132 ± 171	50.69 ± 5.3	1.60 ± 0.06

**Table 4:** Thermomechanical properties of tested samples.

Sample Name	E′30 °C (MPa)	E′50 °C (MPa)	E′75 °C (MPa)	E′130 °C (MPa)	tan δ/Temperature (°C)
PLA	3028	2789	11.98	194.4	2.419/71.65
PLA/CS1	3446	3148	25.2	440.7	1.536/69.74
PLA/CS1.6	3712	3446	32.67	485.6	1.488/70.54
PLA/CS2	3306	3029	35.89	466.4	1.406/71.17
PLA/CS4	3208	2966	28.12	404.1	1.398/69.92

## Data Availability

Data available on request due to restrictions (privacy or ethical). The data presented in this study are available on request from the corresponding author. The data are not publicly available due to the privacy of results belonging to Kazimierz Great University.
